# Awareness of demands and unfairness and the importance of connectedness and security: Teenage girls’ lived experiences of their everyday lives

**DOI:** 10.3402/qhw.v10.27653

**Published:** 2015-06-16

**Authors:** Eva-Lena Einberg, Evy Lidell, Eva K. Clausson

**Affiliations:** 1 School of Health and Welfare, Halmstad University, Halmstad, Sweden; 2 Department of Health Science, School of Health and Society, Kristianstad University, Kristianstad, Sweden

**Keywords:** Adolescents, everyday life, female, gender, lifeworld, phenomenology, sense of coherence

## Abstract

In recent years, a number of studies have demonstrated that stress and mental health problems have increased among adolescents and especially among girls, although little is still known concerning what girls experience in their everyday lives. The aim of this study was to describe the phenomenon of teenage girls’ everyday lives, as experienced by the girls themselves. A phenomenological approach of reflective lifeworld research was used, and the findings are based on eight qualitative interviews with girls aged 13–16 years. The essence of teenage girls’ everyday lives as experienced by the girls themselves can be described as consciousness regarding demands and unfairness and regarding the importance of connectedness and security. The girls are aware of the demands of appearance and success, and they are conscious of the gender differences in school and in the media that affect them. The girls are also conscious about the meaning of connectedness with friends and family, as well as the importance of the security of their confidence in friends and feeling safe where they stay. If teenage girls feel connected and secure, protective factors in the form of manageability and meaningfulness can act as a counterweight to the demands and unfairness of everyday life. For professionals who work with teenage girls, the results from this study can be important in their work to support these girls.

Girls’ living conditions can be important influences on their health and their everyday lives (Larsson, Johansson Sundler, & Ekebergh, 2012). It has been recognized in recent years that girls exhibit more symptoms of illness, such as recurring headaches and nervousness, and feel even more stressed (Hagquist, 2010; Hjern, 2012; Levin, Currie, & Muldoon, 2009; Ravens-Sieberer et al., 2009; West & Sweeting, 2003). Subjective mental health and well-being among adolescents in Sweden have decreased since the beginning of the 1990s (Hagquist, 2010; Hjern, 2012; Petersen et al., 2010). The number of subjective mental health complaints levelled off at the end of the 2000, but is now increasing again (Folkhälsomyndigheten, 2014). Fewer girls report that they are satisfied with life, and the gender gap has grown over the years (Hagquist, 2010; Petersen et al., 2010). These phenomena can be partially explained by individual, family, and school aspects (Bakker, Ormel, Verhulst, & Oldehinkel, 2010; Gillander Gådin & Hammarström, 2003; Hagquist, 1998; Holstein et al., 2009; Saab & Klinger, 2010).

During the teenage years, the need to find one's own identity intensifies according to internal and external changes (Erikson, 1989). A safe and loving family atmosphere and the feeling of importance to one's family (Joronen & Åstedt-Kurki, 2005), as well as stability, open communication, and supportive family relations, are related to adolescents’ life satisfaction, self-esteem, and well-being (Elgar, Craig, & Trites, 2013; Moreno et al., 2009; Rask, Åstedt-Kurki, Paavilainen, & Laippala, 2003; Sweeting & West, 1995). Poor parental relationships and communication and low parental demands are associated with poor general health, feelings of stress, and somatic complaints (Nygren, Bergström, Janlert, & Nygren, 2012; Sweeting & West, 1995). Furthermore, it has been determined that girls are affected by high levels of demands at school and report stress related to concerns about school performance (Eriksson & Sellström, 2010; Murberg & Bru, 2004). Girls’ impaired self-esteem and increased somatic and psychological symptoms from age 12 to 15 are partly explained by the more adverse psychosocial environment that prevails between grades six and nine (Gillander Gådin & Hammarström, 2003). Reduced power, increased demands and, in particular, problems with classmates negatively affect health (Gillander Gådin & Hammarström, 2003).

Marmot (2009) highlights the conditions of everyday life; the circumstances in which individuals are born, grow, and live; and their importance for adolescents’ health. Teenagers’ health is influenced by the circumstances in which they find themselves (Marmot, 2009). Differences in socialization and living conditions can lead to the increased incidence of symptoms in girls (Torsheim et al., 2006). To our knowledge, there are limited reports about how girls 13–16 years of age experience and describe their everyday lives. Teenage girls’ own experiences of everyday life can give insight into their increased mental illness and stress-related symptoms. Knowledge in the form of a deeper understanding of teenage girls’ own experiences is needed so that those who work with and interact with teenage girls can adequately respond to and support them and thereby promote their health. Thus, the aim of this study was to describe the phenomenon of teenage girls’ everyday lives, as experienced by the girls themselves.

## Methods

### Design

An explorative and descriptive qualitative design with a phenomenological approach of reflective lifeworld research was used (Dahlberg, Dahlberg, & Nystrom, 2008). The purpose of a lifeworld perspective is to explore and describe the phenomenon in its most original meaning (Dahlberg et al., 2008). A phenomenon can be understood as a fact or situation as it is perceived or experienced by the subject. In this study, the concrete phenomenon of teenage girls’ everyday lives was studied.

### Participants

To increase the potential for achieving variations in the sample, both public and private schools located in urban and rural areas in the southern part of Sweden were included. Three school nurses received oral and written information about the study and were told that the informants should have a distribution between seventh and ninth grades. The school nurses asked girls (13–16 years of age) if they were interested in participating in the study; 10 girls agreed to participate. One girl became ill, and one conducted interview could not be used because the sound quality was poor. Ultimately, eight interviews were included in the study. Three girls were in the seventh grade, three in the eighth grade, and two in the ninth grade. The informants’ backgrounds varied, and the girls lived with single parent, both parents, parents born in Sweden, and foreign-born parents.

### Data collection

The interviews were conducted in 2006 at each informant's own school in an appropriate room where the interview could be undisturbed. The interviews, lasting between 60 and 90 min, began with a general question: Could you tell me about a typical day? The focus was on their experiences of the everyday life of family, school, and leisure time and the media's representation of teenage girls. The questions were grounded in the lifeworld perspective, and questions for follow-up and clarification were asked depending on how the interview went. All interviews were conducted by the first author (ELE), who is a professional school nurse and has long experience of conversations with teens. Furthermore, all interviews were audio-taped.

### Data analysis

The interviews were transcribed verbatim and read multiple times to become familiar with the material and to capture the whole according to the aim. Each interview was then divided into meaning units to obtain an overview and to enable analysis. Clusters were then made of the meaning units and described in the girls’ own words. Throughout the analysis, there was an open and inquiring approach to the text: How do the informants describe their everyday lives? What variations are there? An attitude of bridling (Dahlberg et al., 2008) was adopted which included openness and respectfulness against the phenomenon, as well as to slow down the process of understanding and wait for the phenomenon to show itself. Bridling includes reflecting upon one's pre-understanding, such as personal beliefs and theories, and to restrain this, so that openness and responsiveness to the phenomenon would prevail. This was carried out by writing down thoughts about the phenomenon and by discussions among the co-authors. The first (ELE) and third authors (EKC) have worked as school nurses. The second author (EL) has experience from research with young women, and the third author has experience from research on schoolchildren's mental health. The first author (ELE) examined the texts multiple times for variations of the phenomenon (Dahlberg et al., 2008). After the first reading of the interviews and clustering of meaning units, discussions were held with the second author (EL) about what emerged in the materials. This led to repeated reading and changes in clustering the meaning units as well as renewed discussions. Throughout the analysis, from the first reading of the interviews and the searching of variations, to the essence of the phenomenon, there was a movement between the whole and the parts and back again to the whole. When the essential constituents were described, the essence emerged against this background, the constituents. The results of the analysis were discussed with the third author (EKC) before the final version was defined to ensure trustworthiness. The essence of the phenomenon is described in the results as a general structure and accompanied by descriptions of the essential constituents to further illustrate the phenomenon. Quotations from the interviews are included to clarify and illustrate the essential constituents of the phenomenon as well as the particularities and nuances of the phenomenon.

### Ethical issues

The study was conducted in accordance with the Helsinki Declaration and was approved by the local ethics committee at Halmstad University (Dnr 90-2005-3444). Information about the study was given both orally and in writing. Written consent was collected from the participating teenage girls and their parents. It was clear that participation was voluntary and could be interrupted at any time and that the information provided would be treated confidentially. Permission to conduct the study was also obtained from the principals of the girls’ schools. To avoid dependencies, informants were not recruited in the municipality where the first author (ELE) was employed as a school nurse. One risk with the study was that the interviews could bring up painful emotions, which is why it was important that, before the interviews began, there was an agreement on where the informants could obtain help and support if the need emerged during the interview. As a result, each school's nurse or social worker was informed about the study and confirmed that it would be possible to refer the girls to them. The interview data were kept in locked storage, and only the first author, who conducted the interviews, could connect the data to the informants; their names were kept separate from the recordings, as well as from the transcribed material.

## Results

The phenomenon of teenage girls’ everyday lives as they experienced them is presented first as a general structure that includes the essential constituents. After this, the essential constituents are described, each with variations on the phenomena that are illustrated by quotations from the interviews. When part of a longer quotation is excluded, it is marked with double slashes (//). Names of places and people in quotes are marked with XXX to ensure confidentiality.

### General structure of the phenomenon of teenage girls’ everyday lives

The essence of teenage girls’ everyday lives as the girls experience them can be described as an awareness of demands and unfairness, and the importance of connectedness and security. The girls are aware of the demands placed on them, and they question the unfairness they perceive that they are exposed to in their everyday lives. They are under pressure to succeed in school, here and now, but also in the future and to become something, not nothing. Demands are also made on how they should act and look to be accepted and loved or well liked. Furthermore, teenage girls experience that boys have more freedom in everyday life and that they are presented differently; where girls often are discussed in the context of negative events, such as violence and ill health, boys are given more space in contexts that are positive. In the connectedness of family and friends, the opportunity is given to receive support and relief from demands. However, the fear of exclusion means that the girls sometimes present false images to their peers in a quest for belonging. Security is important for the girls, and when they feel they can rely on their peers and parents, and even trust their surroundings, they feel that security. Security is threatened by the lack of reliance on anyone and the fear of violence. Analysing the studied phenomenon of teenage girls’ everyday lives reveals an awareness of an existence with many demands and much unfairness but in which the counterbalance is the connectedness and security that the girls care about. The following essential constituents emerged and described in greater detail the phenomenon of teenage girls’ everyday lives: demands; unfairness; connectedness; and security.

#### Demands

Demands in everyday life are related to school and to the girls’ own appearance. Always present in the girls’ everyday lives is performing well, and they often return to this theme in their stories about school and the future. They talk about how they pulled themselves together and prioritize away other things during leisure time to keep up with their schoolwork. They say that without good grades, you cannot achieve anything and that high scores are a prerequisite for a good future. Sometimes, though, they are thinking about whether the demands are realistic and whether the schoolwork they perform will be useful in their futures. Even when they have neither energy nor belief in themselves, there emerges an awareness of the need for good grades. They may be tired after school or in the morning, but they still feel that they must do homework and go to school. The demand for success is also reflected in their expressions of frustration and hopelessness when they do not obtain the opportunities they need to succeed.

I do not care about grades and stuff. Actually the grades are important, but I can't stand studying and stuff like that. You have to have good grades if you are to have a good future, good job and stuff.

Although it can be boring, difficult to work in peace, concentrate, and understand schoolwork, it is clear to the girls that school is important and that it affects their futures. School represents the ability to earn an education that could lead to an interesting profession, a good income, and a position in society. This knowledge is there, even when they do not know what type of education they want or what work they want to do in the future.

Now, I try to pull myself together to take school seriously and so on. If you try it will be more fun, but it might be difficult in school. No, I want to go to … get into secondary school to what I want, and so on. So I do not know what I want to study because I have not thought about it// but I want to have my own business and earn well and have a good time.

The girls often prioritize their school demands over everything else, and some even stop participating in physical activity; they give up their physical activities for the sake of their schoolwork even when they miss the activities. The responsibility for schoolwork weighs more heavily than regular physical activity. “If you have a lot of homework, homework of course comes first, so you have to skip workouts if you have a test and stuff.” Sometimes they feel guilty when they find it difficult to keep up with everything in terms of schoolwork, sports, and socializing with peers. The demands of being a good student can also be about popularity and status among friends; although the girls are aware of the dynamic, it is difficult for them to avoid being drawn into discussions about grades and comparisons between each other.

I think it is because you want to be the best, to be better than your peers and have better grades than them.// I don't know. I also believe that there is something about wanting to be liked.

When they talk about working out and clothing, demands related to appearance emerge. The girls talk about exercise as a way to keep weight off and note that brand-name clothes indicate that you are well-off. The girls also feel that boys place on them the demand to be both stylish and smart. The girls are aware of how they are affected by the media's images of beautiful young women and that these images can lead to low self-esteem. They sometimes compare themselves with the women in the papers, and as one girl put it:

It still affects you, yes; you will not be happier by that and stuff like that, so a lot of pictures … And so, yes, of course I look. if you see a girl who is slim and like that, you kind think you are not good enough and so it's just even more problems.

The demands that the girls mention often affect their behaviour in everyday life. They frequently attempt to adapt, take responsibility, and live up to the demands as they are aware of and questioning the requirements.

### Unfairness

When the girls talk about their everyday lives, they describe that they often are subjected to unfair, negative expectations and a limited and undifferentiated space. Boys are allowed to take more space both at school and in the media. The girls see the power structure that exists around them in their everyday lives, and there is a desire for a more equal life. They reflect on the images conveyed in the media, and they do not always recognize themselves, but they are still affected. When they talk about their experiences with the media, they come to gender roles and gender equality:

For example, that advert, where just, kind of like, the man …, the woman is just type a symbol and it disturbs me. Yes, type, often in soap operas and stuff, yes, the girls are so enormously made up and gorgeous and perfect. Well, they are just like they are not people, the girls, they are only symbols.

In the girls’ descriptions of their everyday lives at school, it appears that boys often obtain more space and that the girls just attempt to adapt. When they are disturbed by boys, they go to group rooms or sit in the corridor so that they can work in quiet. The girls also take responsibility and tell the teacher and help to resolve the situation. “If I'm bothered I should tell the teacher. Then, we have to help each other, if I can sit somewhere else or the boys sit in the corridor and work.” Additionally, it is not only at school that the girls experience how boys take things for themselves at the girls’ expense; it is also during leisure time:

At the youth centre, we usually play pool and table tennis and listen to music and sit and talk. It's fun, although it is very much the boys who do it, the girls not so much.// No, the boys always want to play. The boys are like those who want to decide, decide what girls can and can't do.

At school, the girls are also exposed to patronizing statements from the boys that are not always perceived by the teachers. The girls attempt to explain the boys’ behaviour with their immaturity. The girls also believed that the situation would be better when the boys get older. They try not to bother, but as a girl said “it feels, after all, not funny that people think and say so.”

Unfairness is perceived even when their space at school is restricted because the library and computer lab operating hours do not match their breaks and because everyone is punished if one person does something that is prohibited. Furthermore, the girls experience that the media writes much more about boys and much less about girls. When girls win a competition, it is scarcely mentioned, whereas boys will obtain multiple pages of space. The negative expectations of what girls can achieve compared with boys raises frustration.

You can clearly see that some teachers … you see it really clearly that it's just like guys would be much better at sports than girls.// It's unbelievable, I do not really know but it's just like they're not taking girls’ football seriously, you know.

The girls experience that the media gives a simplistic and negative image of teenage girls as victims of ill health, violence, and drugs. One girl described it this way:

So if there is anything about us teenage girls, it is usually that something, was like rape, or something like that, and I think not, it's boring that it is just boring stuff … that there is never any fun, you know, so we are always subjected to something.

Unfairness may also occur in the home between siblings. One girl told how she helped at home and what her brothers did. She took much of the responsibility and was frustrated that her brothers did not contribute to the same extent. “I'll clean and stuff at home because mom no longer lives with us. Sometimes it's hard and I get angry at my brothers.” According to the girls, unfairness exists in both public and private spaces.

### Connectedness

Connectedness is important for the girls, and therefore, it receives a great deal of attention in their everyday lives. This often involves socializing with friends, but it is also expressed in their stories of family, school, and the Internet. They tell about the support they experience from friends, who listen and care about them, and when they meet their friends, it is primarily for socializing and talking. “It doesn't need to be so much, just getting together and doing something. Sometimes you just sit at someone's home and like, watch a movie and just sit and talk.” Even when school is not otherwise perceived as enjoyable, the positives according to the girls are that they can meet their friends there and that even though there is not much to do during the breaks, they mainly appreciate the opportunity to talk to each other. Connectedness in school is especially important when the girls are geographically far from peers during their leisure time. However, it can be difficult to engage in conversation during breaks at school if they also do not see each other during leisure time. “Yes, sometimes you feel that it is tough and so you want to get there, because of the talks.” They describe sports activities during leisure time as an opportunity for connectedness, and they also talk with their peers so as not to lose contact after compulsory school. The importance of connectedness is also expressed in the fear of exclusion, which can lead them to act differently to be liked.

I think you sometimes make a fuss to be liked// afterwards you're thinking -oh well, why did I do that? Why am I not just myself?// you think, -oh well, then I will not be the most liked by that person; you still get the idea that you're left outside.

Family is important whether everyone in the family is near or some family members live far away. They often talk appreciatively about their families, what they do together and how it is important. Doing things with their parents creates the opportunity to build good relationships with them for the future. As one girl reported:

Yes, we actually do a lot, it's fun. You want to have a good relationship with your parents and stuff, so that you dare to talk to them about things. If something like that would have happened and you wonder anything, so you have a good relationship with them.

The importance of connectedness can also be expressed in their dissatisfaction with their origin families and their desire to live with someone else, and the girls report that it can feel empty at home when their siblings leave home. One girl expressed that she felt happy when all family members were happy and gathered at home. Another girl reflected over the Internet's opportunities for social contacts and connectedness:

There may be some friends I don't have here at school, or some live in XXX, who were my friends when I was at my previous school. Yes, instead of letters and stuff, yes you reach each other more easily.

The girls feel that they can talk with their friends all the time whether they meet face to face or not, but it is also fun to sit together in front of the computer. The Internet is experienced primarily as something positive, with reference mainly to the ability to connect with friends, both those who live in the same city and those who are far away.

### Security

It emerges in the interviews that security is an important factor in the girls’ lives. It is about trust in friends and parents but also feeling safe at school and during leisure time, both outdoors and in others’ homes. Security affects the girls’ relationships with others and their freedom of movement. They talk not only about the security that close friends can provide but also about experiencing difficulty having confidence in their peers. “Oh it can be hard sometimes, you know, that there is bullshit that you have in school. We girls are really good at talking shit, you never know what is being said behind your back.” Peers pretend that they are good friends and then break the trust they have created. If the girls have someone they trust who they can tell everything to, it can give a sense of relief. Security is also about daring to talk to their parents and asking them for help. One girl explained:

If something happens … I call her (mother) rather than being afraid that she will get angry and stuff. It feels really nice and good. For many, they need to lie to their parents when they are going off to parties and stuff.

Sometimes, however, reliance on adults is missing, as was demonstrated in the expression “I do not like to talk to adults because they do not understand.” There is a resignation towards adults, and peers may instead represent security. Peers provide security both at school and in the evenings and on weekends. The girls feel safer when they are with their friends when they go to parties, and they avoid going alone. “It's safer, like, you know your friends and you will be together with them, it feels just, like, good. It's hard to say what it is that feels good, it feels good.” If they live in an area where everyone say hello to each other, it feels safe. The violence in society that the media reports, in contrast, creates uncertainty and fear. The girls sometimes do not dare to go out because of fear of being subjected to violence, and when they are at friends’ parties, they are aware of the risk that uninvited persons may appear simply to cause trouble. It is safe if everyone knows each other and if they can quickly reach a parent via mobile phone. “Mom always said, if anything happens, just call and I'll pick you up, so I feel confident that there is always someone who can pick me up any time of day.” Security for the girls entails both security in their social relationships and also physical security in the form of feeling safe where they stay, both indoors and outdoors. These girls attempt to avoid threats of violence by seeking support from each other.

## Comprehensive understanding

According to the teenage girls’ lived experiences, we understand that the girls are aware of the gender order that exists in society. Connell (2003) means that both girls and boys are growing up in the shadow of society's gender order, and that is how they learn about masculinity and femininity in the adult world. Children are not passive recipients of gender norms but they must relate to them, and it is difficult to break patterns that you have grown up with. The inequalities and injustices of gender order are often to men's and boys’ advantage, although not all men and boys benefit from the prevailing order (Connell, 2003). The teenage years are a time of change which may create stressful experiences (Erikson, 1989). For teenage girls, it also means discovering gender-related unfairness in society. The girls’ awareness of demands and unfairness involves the awareness of the gender order. The girls see the gender-related unfairness in the society and question them but at the same time it is difficult for them to defend themselves against it such as the demand on appearance and the unfairness that allows the boys to dominate over the girls in school. According to Connell (2003) even if they are aware of the gender order, it is difficult for them to not follow existing pattern and structure in the society. How the girls understand and manage the demands and unfairness they experience can be further explained by Antonovsky's (1991) salutogenic model with the concept of sense of coherence (SOC) and its components; comprehensibility, manageability, and meaningfulness. *Comprehensibility* refers to the experience that things are predictable and understandable, and *manageability* concerns the feeling that there are resources you can use to meet the demands. *Meaningfulness* stands for participation and also that some things in life are emotionally important (Antonovsky, 1991). The use of SOC and its components in this interpretation was chosen because the model deals with how people understand and manage the situation they are in and because of the salutogenic approach which brings focus on resources that can support the girls in their everyday lives. In relation to SOC, and the components comprehensibility and manageability, the girls in the current study predict that their grades affect their futures and they manage this by taking great responsibility for their schoolwork. The girls understand their situation as they need to perform better than others to have the same chance to succeed. They work hard at school so that they will have a better standing when they compete for work later in life, but this dedication perhaps comes at the price of their health, given that stress and illness are associated with girls’ stress over schoolwork (Eriksson & Sellström, 2010; Nygren, 2007). According to this, it seems the girls need more resources to deal with the stress of schoolwork that they experience. Resources could be in the form of support from adults at school or at home, and how to handle the schoolwork. Furthermore, the girls are aware of appearance-related demands but find them difficult to manage. They understand that the images of beautiful people are not always realistic but at the same time they experience that they should look like this. Perhaps, more thorough discussions together with the girls about this phenomenon could give the girls resources to manage it and thus increase the comprehensibility and manageability. In relation to SOC and the component manageability, the girls try to manage the unfairness, sexual harassment, and violence, they experience in their everyday lives through the security parents and friends can offer. If this unfairness is comprehensible for the girls and can be discussed, the girls try to understand but also to question it, why it seems that it is not always comprehensible for them. On the other hand, and in relation to SOC and meaningfulness, the girls’ experience of connectedness allows for participation and support as well as understanding, which help the girls to manage demands and unfairness.

Antonovsky (1991) believed that SOC, how a person views the life, develops through childhood until the end of young adulthood and then becomes a stable dispositional orientation. According to Antonovsky, environment, culture, and norms influence experiences that an individual encounters in life, which in turn creates a stronger or weaker SOC (Antonovsky, 1991). With this in mind, the girls’ experience of their everyday lives may affect the development of their SOC. Studies have also found that adolescent girls between 15 and 18 years have lower level of SOC than boys of the same age (Rivera, Garcia-Moya, Moreno, & Ramos, 2012). Furthermore, it has been found that support from family members, a positive climate in family relationships, and support from peers promote SOC. There are also indications that the neighbourhood can affect the development of SOC (Rivera et al., 2012). In relation to this, the girls’ experiences of connectedness and security can be seen as factors in the girls’ environment that promote the development of SOC and also as protective factors related to the girls’ health and well-being.

## Discussion

The results show that the essence of teenage girls’ everyday lives as experienced by the girls themselves can be described as awareness about demands and unfairness and about the significance of connectedness and security. Awareness about demands such as from school and the media was expressed by the girls. Even though their peers were important, they often prioritized schoolwork over everything else, and similar results have been found in other studies. According to Nygren (2007), girls study as frequently as they can, and a number of studies have shown that girls are stressed over schoolwork (Eriksson & Sellström, 2010; Murberg & Bru, 2004; West & Sweeting, 2003). Girls earn better grades at school, but they also experience poorer health compared with boys (Eriksson & Sellström, 2010; Nygren, 2007). Girls often take more responsibility than boys, and both boys and girls relate this to gendered life circumstances (Landstedt, Asplund, & Gillander Gådin, 2009). Boys have been favoured, and they ultimately also earn better wages. Assuming theories of the gender power structure (Connell, 2003), girls may not trust that society equitably assesses their skills, and therefore, it is too risky for them to take less responsibility regarding schoolwork (Landstedt et al., 2009). It appears that girls’ education strategies are affected by society's gender power structure (Landstedt et al., 2009; Nygren, 2007). All girls in the present study were aware that grades affected their futures, and the need to be successful emerged as part of their everyday lives. Among the requirements for education and success were also the expectations that they should take more space for themselves in society. According to Holm (2008), it appears today that young people are expected to broaden their repertoires, to be successful at all levels and to be everything (Holm, 2008).

The girls in this study were also aware of the media's influence, but they found it difficult to defend themselves against the media's images of what women should look like. The media's impact on girls has also been described in other studies (Banister & Schreiber, 2001; Chow, 2004; Tiggemann & Miller, 2010). Beauty ideals and norms affect body image, which in turn influences self-esteem, and many girls are unhappy with their appearance (Lunde, Frisén, & Hwang, 2007; Meland, Haugland, & Breidablik, 2007). One's appearance is a part of one's body, and from a lifeworld perspective (Dahlberg et al., 2008), it is through the body that we have access to the lived world, the lifeworld. The body's importance cannot be overlooked, and according to Merleau-Ponty (2000), we become aware of our own bodies through our experiences with the world and we gain access to the world through our bodies. Our bodies are constantly with us, and by meeting others, I become aware of my body, and my identity is shaped by my lived body and by being to the world (Merleau-Ponty, 2000). Young (2000) argues that early on, girls are limited by the prevailing norms about what they should be and what they can do, which affects their relationships with their bodies and their self-esteem. Body image is affected by the tension between being both subject and object (Young, 2000).

Unfairness in everyday life was described by the girls; they expressed that boys were allowed to take up more space at school. Previous studies have found that the gender inequality in school favours boys, that boys dominate over girls and verbally take a lot of space at the girls’ expense, and that girls are aware of this (Gillander Gådin & Hammarström, 2005; Nygren, 2007). Girls take responsibility and maintain order in classrooms (Gillander Gådin, Weiner, & Ahlgren, 2013). It has been found that girls blame themselves for this (Nygren, 2007), which we did not find in the present study. These girls, rather, were attempting to adapt and to find explanations for why boys took more space, for example that boys are immature; similar findings were also noted by Holm (2008). The present study also reveals that girls in their everyday lives are often subjected to degrading and demeaning comments. Sexual harassment is a problem at school (Carlerby, Viitasara, Knutsson, & Gillander Gådin, 2012; Witkowska & Menckel, 2005), and the experience of harassment is associated with impaired health and well-being (Bakker et al., 2010; Bucchianeri, Eisenberg, Wall, Piran, & Neumark-Sztainer, 2014). It increases the risk of symptoms such as nervousness, anxiety, and worry in girls (Gillander Gådin & Hammarström, 2005). Violations are sometimes excused as jokes or immaturity, with the result that the problem is not taken seriously and power aspects are not addressed (Holm, 2008). According to Swedish law (SFS 2010:800) and the Swedish National Agency for Education (2011), the schools have an important role in making prevailing norms visible and in ensuring that boys’ and girls’ actions and relationships are valued equally. The current study also revealed that unfairness is perceived not only at school but also in private, which has also been observed in other studies. The girls perform more housework than their brothers (Nygren, 2007) and experience greater demands from their parents regarding school performance and their leisure time (Nygren et al., 2012). The girls in this study are frustrated that they have been subordinated and must adapt and make sacrifices. The unfairness that girls experience in their everyday lives and their questioning of it give the impression that they are aware of the gender power relations that exist in society. They attempt to manage the unfairness at school by stating that boys are immature and by adapting to the situation. To support girls, gender equality should be emphasized in schools and communities, and it should be a priority in accordance with Swedish law (SFS 2010) and regulations (Swedish National Agency for Education, 2011). Awareness of demands and unfairness can perhaps be both good and bad. It can push the girls to challenge the power structure, but it can also lead to frustration and ill health.

Connectedness in everyday life emerged as important for the girls in this study and is in line with other studies in which good relationships with family and friends have been associated with girls’ well-being (Moreno et al., 2009; Wiens, Kyngäs, & Pölkki, 2014). After schoolwork, the girls spent most of their time with friends. They are aware of the support close friends can provide but also aware of the fear of alienation, which makes them sometimes disguise themselves to be liked. Relationships with peers are more important during adolescence than they are earlier in life, and exclusion is therefore very difficult to accept (Hwang & Nilsson, 2011). Young people themselves relate conflicts with peers, bullying, and concerns about criticism from others to their own health (Hendry & Reid, 2000; Løhre, Lydersen, Paulsen, Mæhle, & Vatten, 2011) and feel that support from peers makes it easier to address problems (Hendry & Reid, 2000; Wiens et al., 2014). The awareness of the school's role as an arena for connectedness was also obvious for the girls. Although peers appear to be the most important, there is also awareness about the importance of family in the girls’ narratives. The importance of family and friends and their influence on health have also been found in previous research (Brolin Låftman & Östberg, 2006; Landstedt et al., 2009; Larsson et al., 2012; Wiens et al., 2014). In connection with this, the school staff have an important role both in supporting good relationships between pupils, but also involving parents in their efforts to promote girls health.

Security affected the girls’ everyday lives both at school and during leisure time, and they therefore sought support from each other, both to feel secure socially and also to reduce the risk of becoming victims of violence. The girls were aware that there are risks of walking alone in certain places, with the result that they either failed to go out or took care not to be alone. Previous research has shown that many young women have experienced violence or threats of violence (Banister & Schreiber, 2001; Landstedt & Gillander Gådin, 2011). The girls in this study also described that the media's reporting on violence creates insecurity. Safety and security are important to health, whereas violence or threats of violence increase the risk of ill health (Hensing & Hammarström, 2008). In the current study, the girls expressed that security entails having confidence in peers and daring to tell everything can give a sense of relief. Furthermore, reliance on parents or other adults gave security. Other studies have also described that relationships with others can give strength and safety (Hendry & Reid, 2000; Larsson et al., 2012), and it is known that their relationships with their parents are important for young people's health and welfare (Brolin Låftman & Östberg, 2006; Joronen & Åstedt-Kurki, 2005; Nygren et al., 2012). In connection with the above, there is a need to increase security for girls where they meet and socialize. Staff who meet girls should be aware of the need for security for the girls and their parents’ role in this.

## Methods discussion

Teenage girls’ everyday lives are a complex phenomenon; therefore, a qualitative design with a phenomenological approach of reflective lifeworld research with individual interviews was chosen such that experiences, through feelings and thoughts, would be revealed. These results are based on interviews that were conducted in 2006, which could be considered a long time ago. However, girls’ expressions of mental illness have not decreased; they have, rather, increased, and there are few studies in which girls themselves have had the opportunity to express how they experience their everyday lives. This demonstrates that there is still a need for deeper understanding in the area and could mean that this study's results are still relevant. Eight interviews can be considered a low number, but from a lifeworld perspective, the variations in the data are more important (Dahlberg et al., 2008). The girls varied according to family situation, school affiliation, and place of residence. It was important that the informants felt safe so that they would share their experiences, and it was carefully stressed before the interviews that the interviewer had no connections to their schools. During the interviews, the feeling was that the girls felt comfortable with the situation and were pleased to talk about their everyday lives, which likely contributed to the diverse and reflective responses. For informants, it can be a positive experience that someone wants to listen to them (Kvale & Brinkmann, 2009). The girls were open and honest in their stories of their everyday lives. They told about events and situations from their everyday lives and gave rich descriptions of their feelings. The first-author's preconceptions as an active school nurse could have been a good foundation for the study but could have also affected her understanding of the phenomenon. During the interviews, the first author deliberately worked to be open and curious in the meetings with the girls. Openness to what emerged was also evident in the analyses, and the fact that two other authors were involved in the analyses increases the trustworthiness.

In studies with a lifeworld perspective, the researcher's ability to be open, receptive, and responsive to what emerges is crucial to the outcome (Dahlberg et al., 2008). During the analysis and the process of understanding, a bridling attitude was adopted which meant that the authors questioned their own thoughts and interpretations. Girls’ stressful daily lives’ were part of the first author's preconceptions, but this study revealed that many girls felt unfairly treated, which was partly a new perspective. It was unexpected that they appeared to be so aware of and frustrated by unfairness, and that all of them felt this way irrespective of their backgrounds was surprising. This surprise could suggest that openness prevailed in the interviews. In addition to the co-author's discussions during the process, the study's credibility is also reinforced by the included quotations, which make it possible for the reader to value how the informants expressed themselves.

According to Dahlberg et al. (2008), results from phenomenological studies can be generalized but must be observed in context; data analysis leads to a general structure, or, in other words, an essence of the phenomenon. In this study, the results regard teenage girls who lived in Sweden. The girls interviewed attended different schools and classes and lived in both urban and rural areas with diverse family situations. These results could be useful in areas and activities in which teenage girls are encountered, for example, in health care and in educational and recreational activities in cultures that are similar to Sweden's. Finally, it is important to note that teenage girls are not a homogeneous group, so this study has limitations. How the girls felt about their daily lives based on ethnicity and social class was not addressed in this study.

## Implications

Personnel who work with or otherwise come in contact with teenage girls can use these results to gain a better understanding of these girls’ everyday lives. Increased understanding from a gender perspective is needed to support girls more adequately, given that symptoms of ill health can be expressions of the prevailing gender power structure. It is also important to note that security and connectedness are factors that can promote health and well-being. Furthermore, open and reflective dialogue with teenage girls can lead to deeper understanding of their situations. This study focused on teenage girls’ everyday lives, but it would also be of interest to gain in-depth knowledge concerning teenage boys’ everyday lives and to compare the two, including how social structures and processes affect their everyday lives. Increased knowledge of young people's everyday lives can facilitate and promote processes that are important for their health and well-being.

## Conclusions

The essence of teenage girls’ everyday lives as they experience them can be described as an awareness of demands and unfairness, and the importance of connectedness and security. Teenage girls experience everyday life demands to succeed both in school today and in the future. They are also aware of the demands for their appearance and the unfairness in that boys obtain extra space in more positive contexts. Connectedness with peers is important, and the girls are aware of the support that friends can provide but also of the importance of their relationships with their parents and families. The girls are also conscious of what security means in terms of trust in others and of the results of fear of violence, particularly that they dare not stay anywhere. Connectedness and security can be important counterbalancing factors to the demands and unfairness that the girls’ surroundings convey to them. If teenage girls feel connected and secure, protective factors in the form of manageability and meaningfulness can act as counterweights to the demands and unfairness of everyday life. Despair regarding and resignation to life's demands and unfairness may lead to feelings of insecurity and alienation.

## Authors' contributions

E.-L. Einberg conducted the interviews with the girls and had primary responsibility for the analysis and for writing the manuscript. E. Lidell and E.K. Clausson contributed to the analysis and the manuscript writing.
